# Supplementary material for: A locally solvent-tethered polymer electrolyte for long-life lithium metal batteries
Source: Nat Commun. 2024 May 9;15:3914. doi: 10.1038/s41467-024-48078-7 (PMC11082227; doi:10.1038/s41467-024-48078-7)
Supplement: Supplementary file 1 — Supplementary Information [file 41467_2024_48078_MOESM1_ESM.pdf]

A locally solvent-tethered polymer electrolyte for long-life lithium metal batteries

Yanfei Zhu[1,#], Zhoujie Lao[1,#], Mengtian Zhang[1,#], Tingzheng Hou[1,*], Xiao Xiao[1], Zhihong Piao[1], Gongxun Lu[1], Zhiyuan Han[1], Runhua Gao[1], Lu Nie[1], Xinru Wu[1], Yanze Song[1], Chaoyuan Ji[1], Jian Wang[2], and Guangmin Zhou[1,*]

[1] Tsinghua-Berkeley Shenzhen Institute & Tsinghua Shenzhen International Graduate School, Shenzhen, 518055, P. R. China

[2] Canadian Light Source, Saskatoon, S7N 2V3, Canada

[#] These authors contributed equally.

[*] Correspondence to tingzhenghou@sz.tsinghua.edu.cn, guangminzhou@sz.tsinghua.edu.cn.

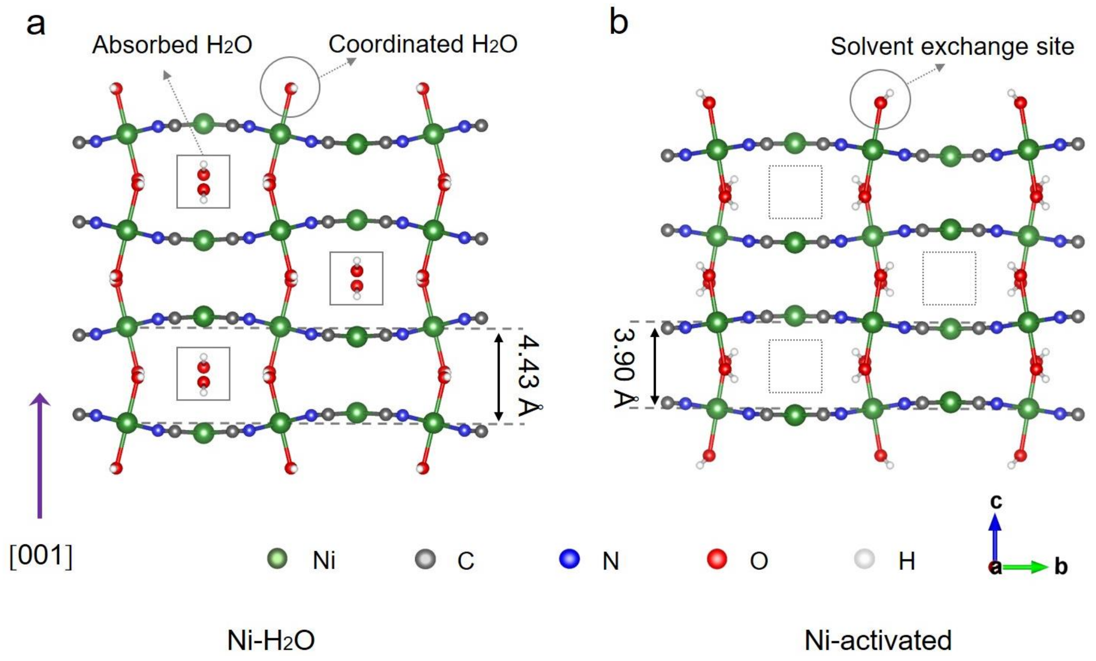

Supplementary Fig. 1 | Front view of the crystal structures of (a) Ni-H_2O and (b) Ni-activated.

The Ni-activated sample was obtained by a thermal dehydration treatment on the Ni-H_2O sample with internal channels created.

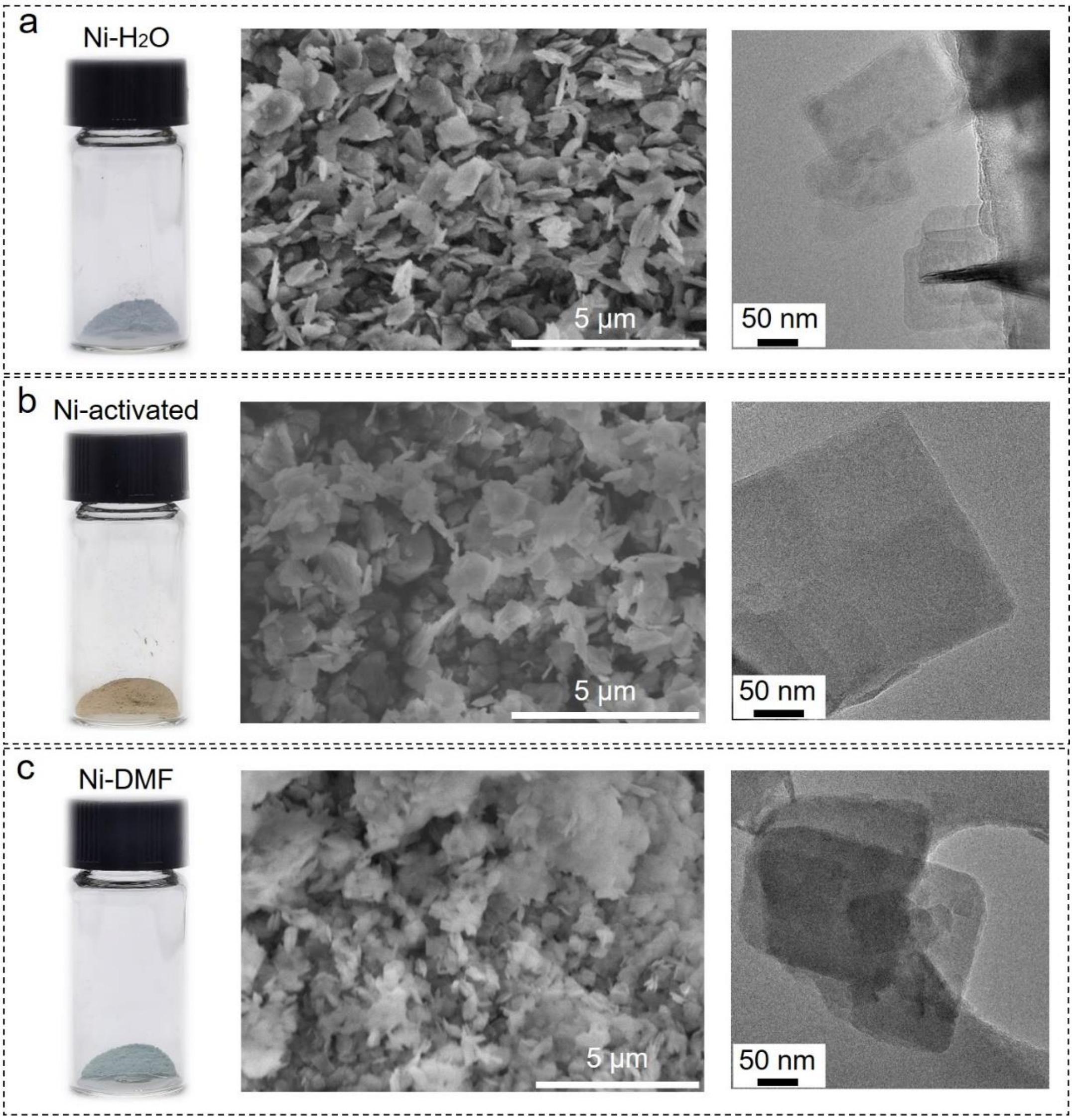

Supplementary Fig. 2 | Digital, SEM, and TEM images of (a) Ni-H_2O, (b) Ni-activated, and (c) Ni-DMF. The samples demonstrate obvious color change during preparation processes.

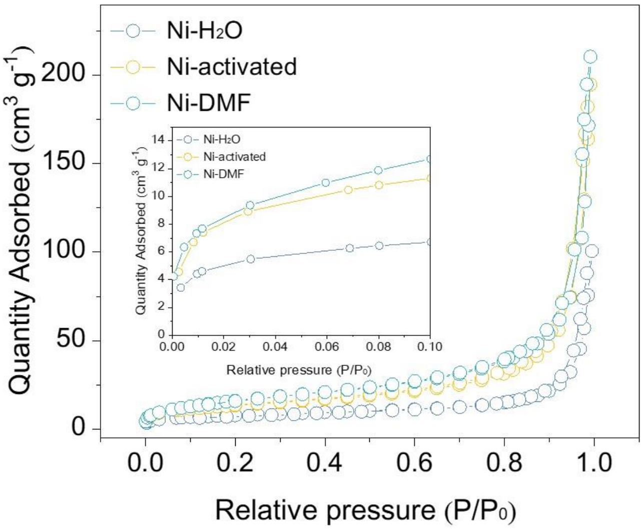

Supplementary Fig. 3 | N_2 adsorption/desorption isotherms of Ni-H_2O, Ni-activated, and Ni-DMF. The inset is the magnified image at the selected relative pressure range from 0 ~ 0.1.

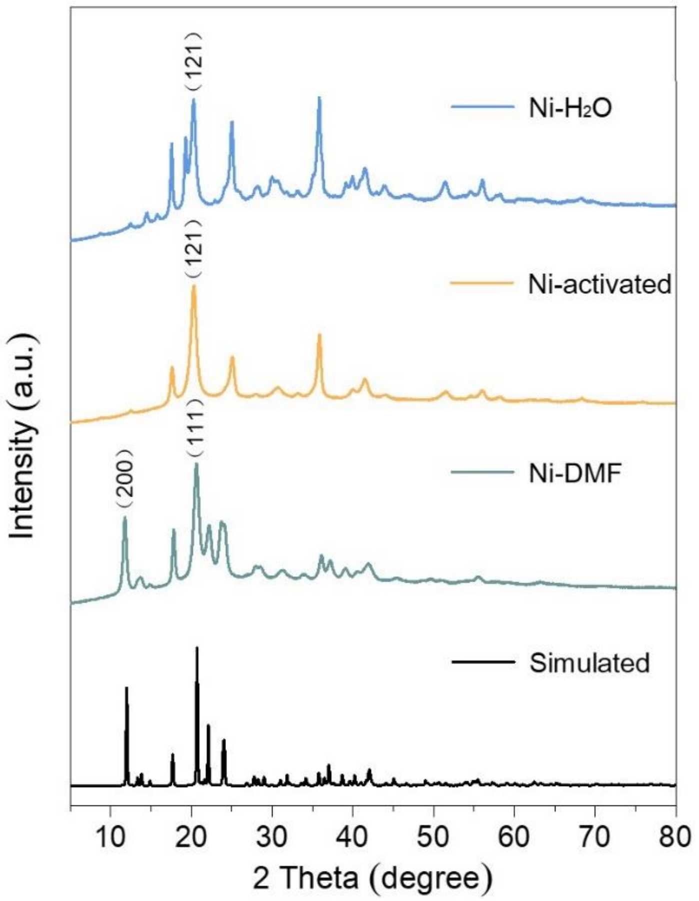

Supplementary Fig. 4 | Powder X-ray diffraction results for Ni-H_2O, Ni-activated, and Ni-DMF.

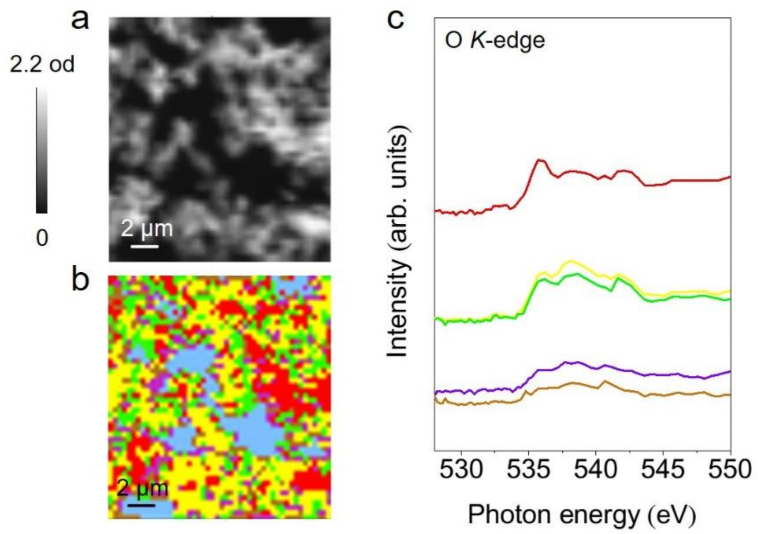

Supplementary Fig. 5 | Scanning transmission X-ray microscope (STXM) results of Ni-H_2O. (a) STXM stack average optical density (OD) image and (b) corresponding color-coded spatial distribution of O containing species; (c) O *K*-edge spectra of the corresponding color-coded regions in (b). For example, the red line is the O *K*-edge spectrum which is collected at the red region in (b).

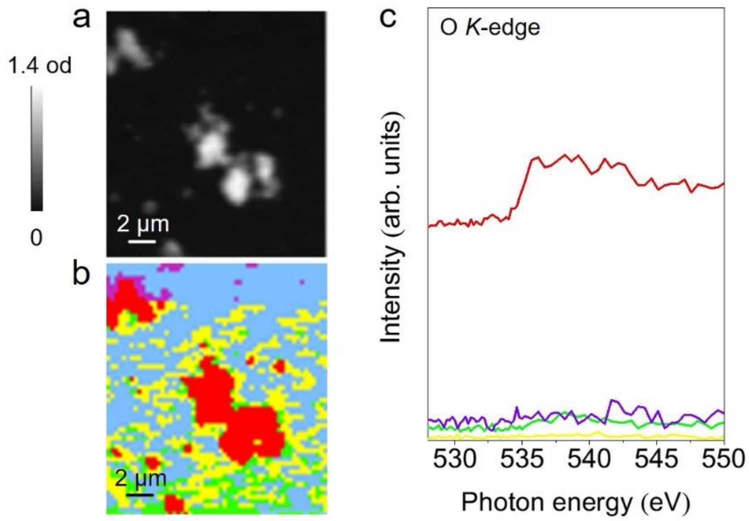

Supplementary Fig. 6 | STXM results of Ni-activated. (a) STXM stack average OD image and (b) corresponding color-coded spatial distribution of O containing species; (c) O *K*-edge spectra of the corresponding color-coded regions in (b). For example, the red line is the O *K*-edge spectrum which is collected at the red region in (b).

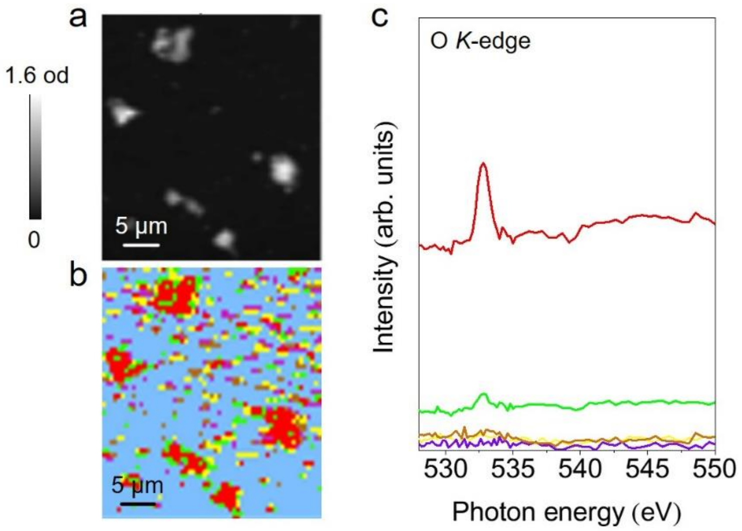

Supplementary Fig. 7 | STXM results of Ni-DMF. (a) STXM stack average OD image and (b) corresponding color-coded spatial distribution of O containing species; (c) O *K*-edge spectra of the corresponding color-coded regions in (b). For example, the red line is the O *K*-edge spectrum which is collected at the red region in (b).

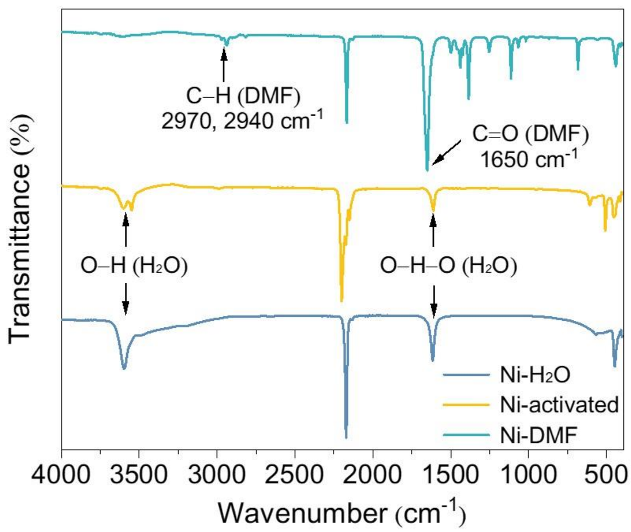

Supplementary Fig. 8 | Attenuated total reflectance-Fourier transform infrared (ATR-FTIR) spectra of Ni-H_2O, Ni-activated, and Ni-DMF.

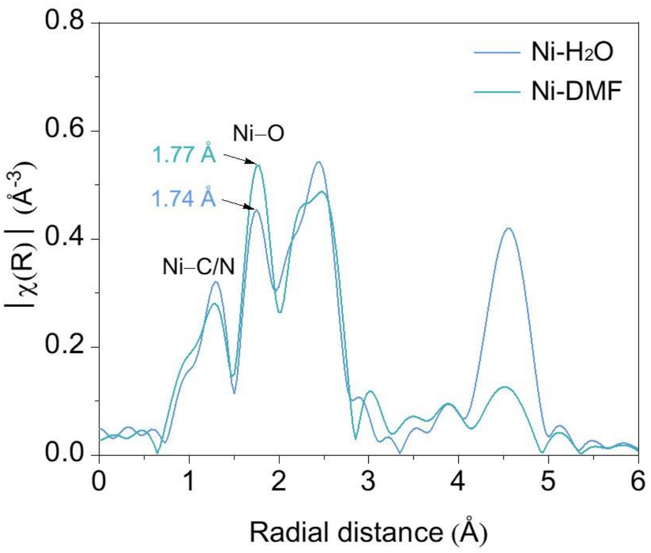

Supplementary Fig. 9 | k^3-weighted Fourier transform of extended X-ray absorption fine structure analyses for Ni-H_2O and Ni-DMF.

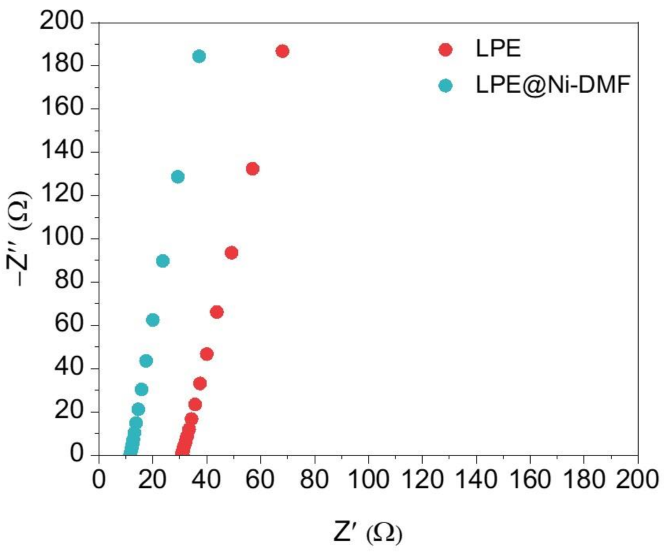

Supplementary Fig. 10 | Nyquist plots of LPE and LPE@Ni-DMF at room temperature.

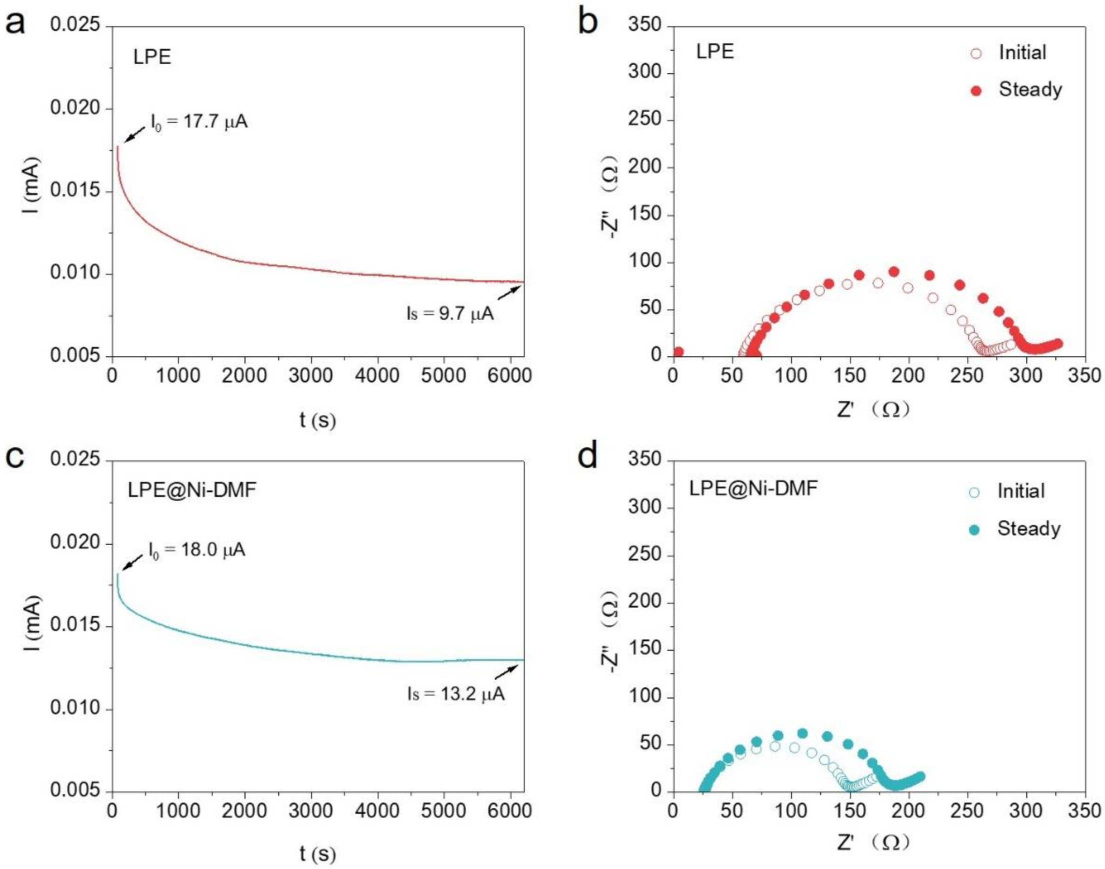

Supplementary Fig. 11 | Current evolution of Li||Li cell using (a) LPE and (c) LPE@Ni-DMF under a polarization voltage of 10 mV and the related Nyquist plots (b) and (d) before and after test.

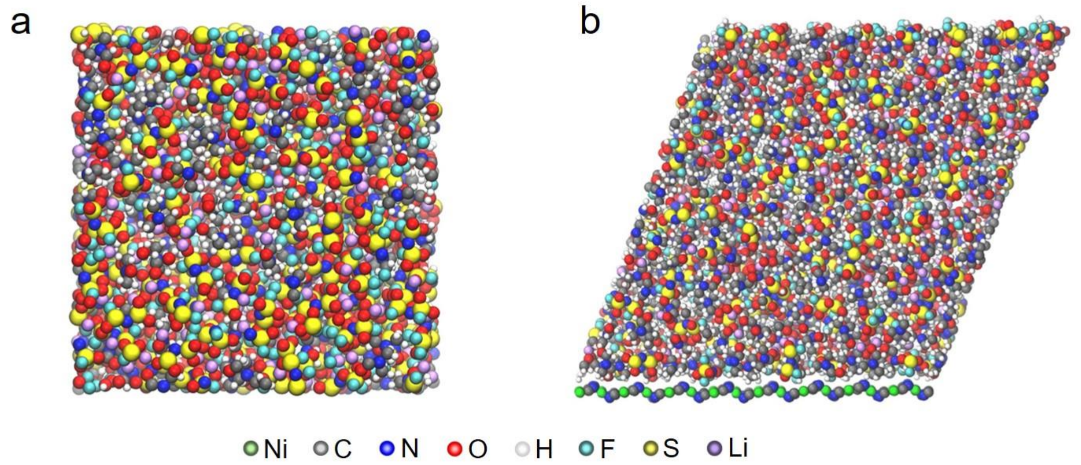

Supplementary Fig. 12 | Molecular dynamics (MD) simulation boxes (a) without and (b) with Ni-DMF. LPE was modeled with 192 DMF, 264 LiFSI to obtain an effective concentration of 7.15 M. The atomic position obtained from single crystal X-ray diffraction of LPE@Ni-DMF was used as the initial structure to build a supercell containing 128 Ni, 128 CN, 512 DMF, and 180 LiFSI.

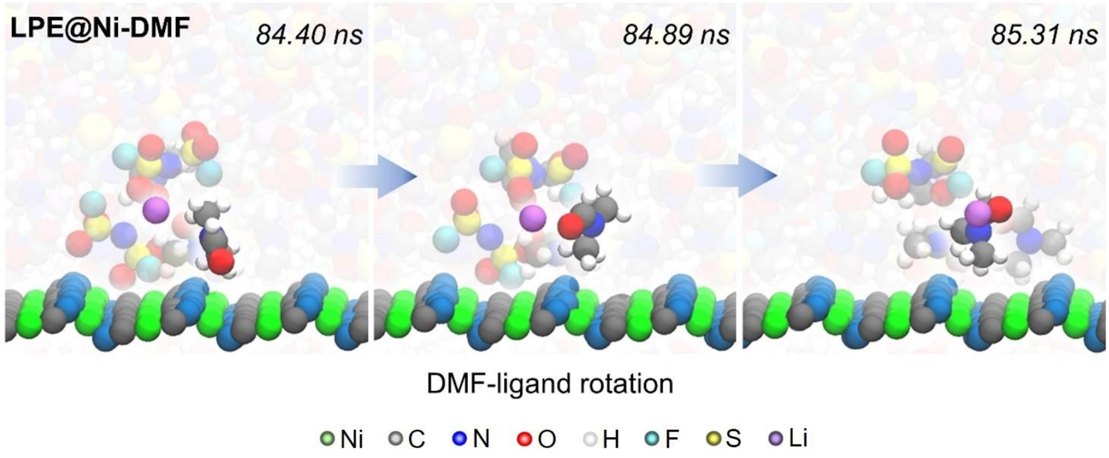

Supplementary Fig. 13 | Snapshots of MD trajectories showing local motion of the DMF ligand during Li^{+} transfer. The snapshots from 84.40 ns to 85.31 ns elucidate that not only the horizontal translational motion of Li^{+} but also the rotational dynamics of the DMF ligands.

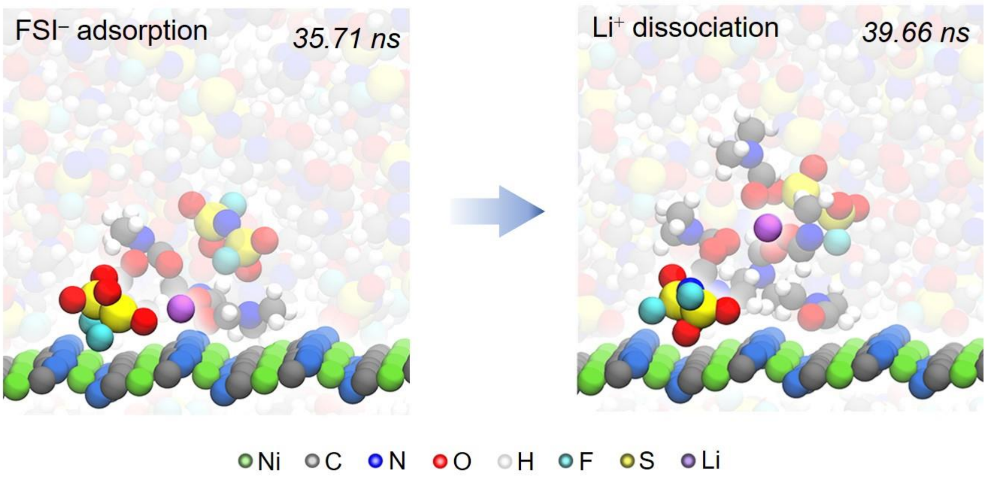

Supplementary Fig. 14 | Snapshots of the MD simulation of LiFSI dissociation. The snapshots from 35.71 ns to 39.66 ns elucidate that FSI^{-} can be immobilized onto the Ni-DMF framework and the motion of the paring Li^{+} can thus be uncorrelated from the FSI^{-}.

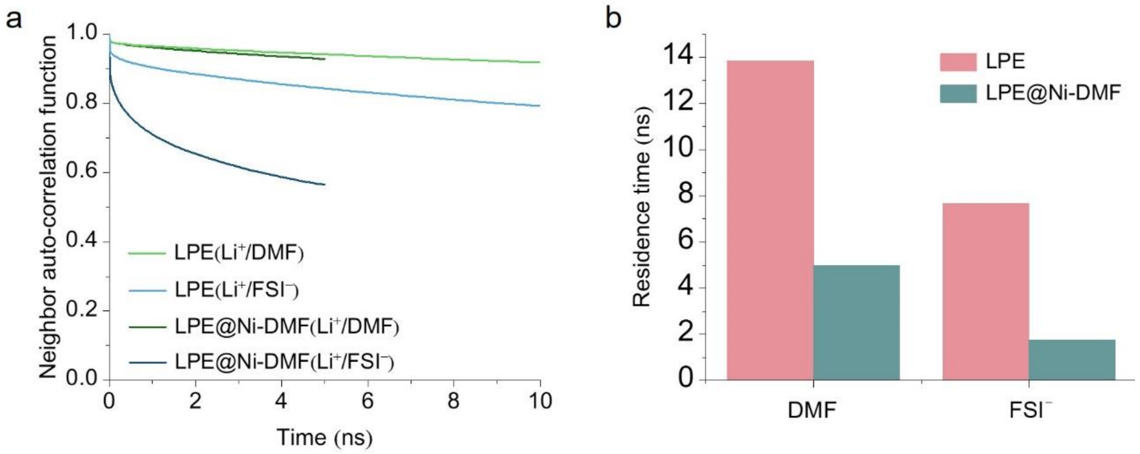

Supplementary Fig. 15 | Neighbor auto-correlation function for Li^+/DMF and Li^+/FSI^- ion-pairs in LPE and LPE@Ni-DMF (a) and corresponding residence time comparison (b).

The residence times of Li^+-X pairs (Li^+-DMF and Li^+-FSI^-) are calculated by computing the lifetime correlation function:

$$P_{Li^+\text{-}X}(t) = \langle H_{Li^+\text{-}X}(t) \cdot H_{Li^+\text{-}X}(0) \rangle \qquad (1)$$

where $H_{Li^+\text{-}X}(t)$ is one if Li^+ and X are neighbors at time t and zero otherwise.

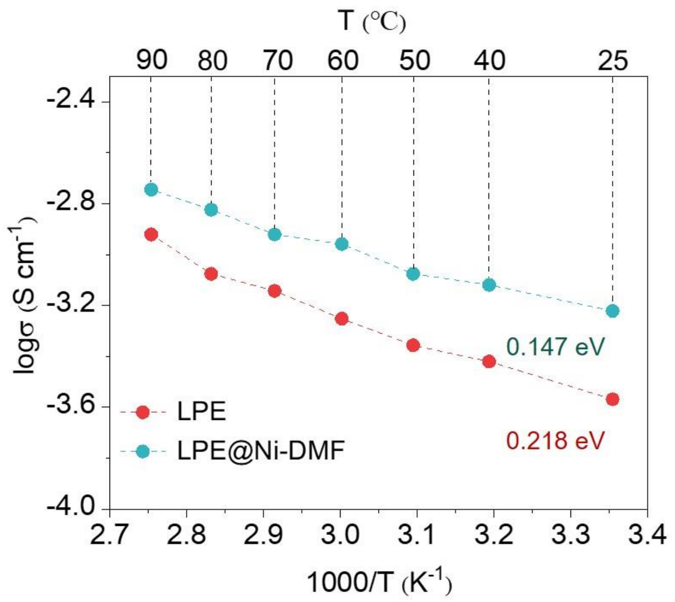

Supplementary Fig. 16 | Arrhenius plots of LPE and LPE@Ni-DMF at different temperatures.

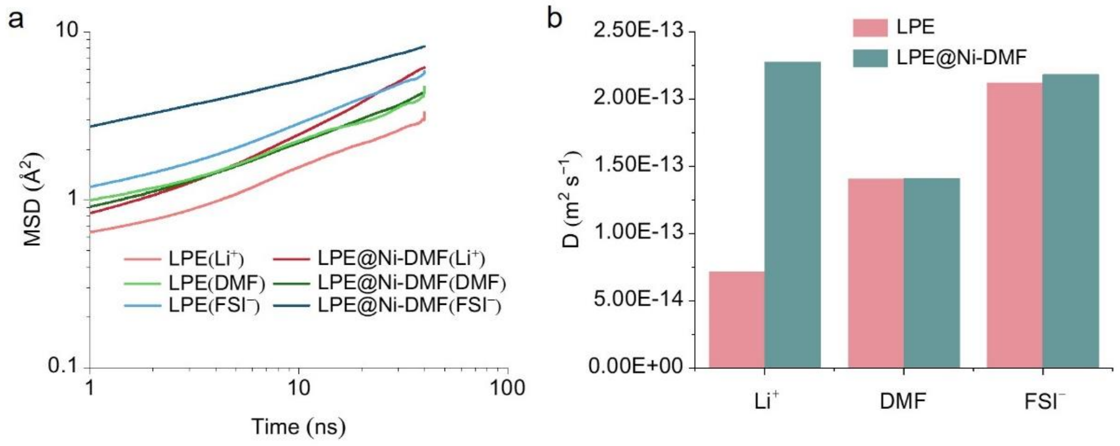

Supplementary Fig. 17 | The diffusion behavior of Li^+, DMF, and FSI^- in LPE and LPE@Ni@DMF. (a) Mean square displacements (MSD), and (b) self-diffusion coefficients (D).

The self-diffusion coefficients were extracted from the MD simulation by analysis of the mean square displacement (MSD, $\langle(\delta r)^2\rangle$) over time. The slope of the linear regime in the MSD was obtained for each simulation duration of 1 ns and averaged over at least 10 ns of the production runs to obtain:

$$D = \frac{1}{6}\lim_{t\to\infty}\frac{d}{dt}\langle(\delta r)^2\rangle \qquad (2).$$

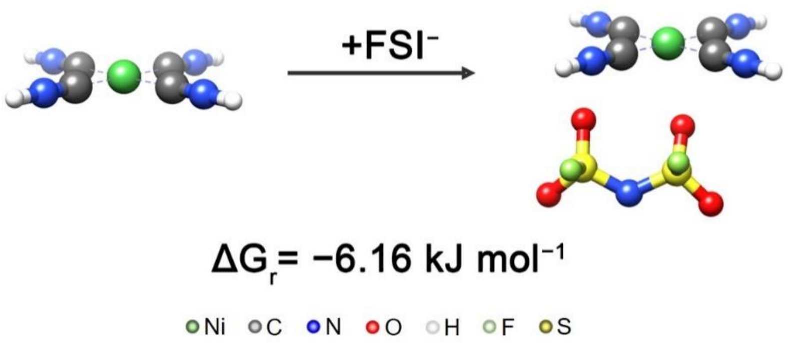

Supplementary Fig. 18 | Calculated adsorption Gibbs free energy of FSI^- anion on $[Ni(CN)_4]^{2-}$ site.

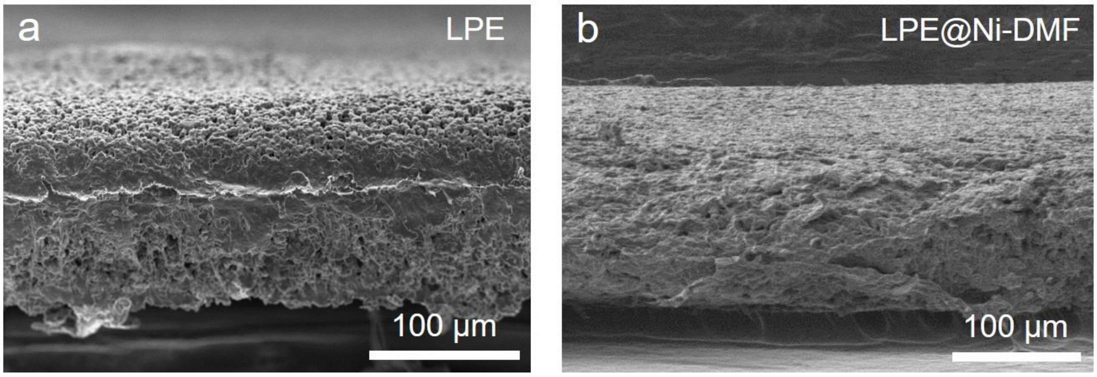

Supplementary Fig. 19 | SEM images of (a) LPE and (b) LPE@Ni-DMF membranes.

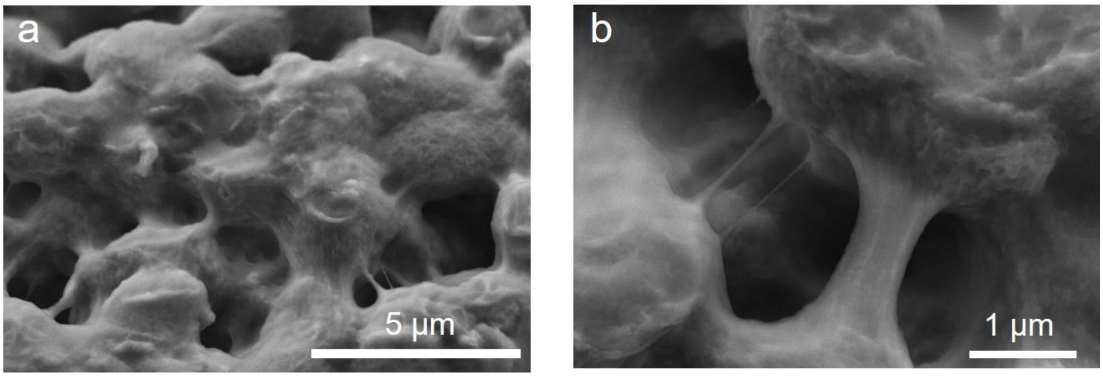

Supplementary Fig. 20 | SEM (a) and corresponding magnified (b) images of the internal architecture of LPE. It can be seen that particles interconnect with each other without Ni-DMF filler embedded.

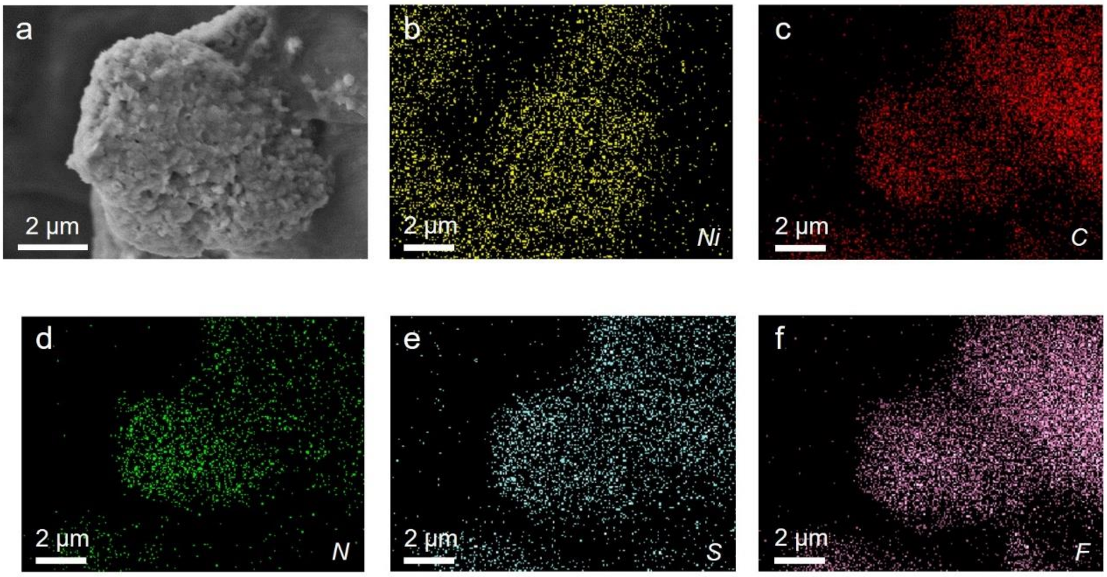

Supplementary Fig. 21 | Magnified SEM image of LPE@Ni-DMF (a) and corresponding energy dispersive spectrometer mapping (b-f).

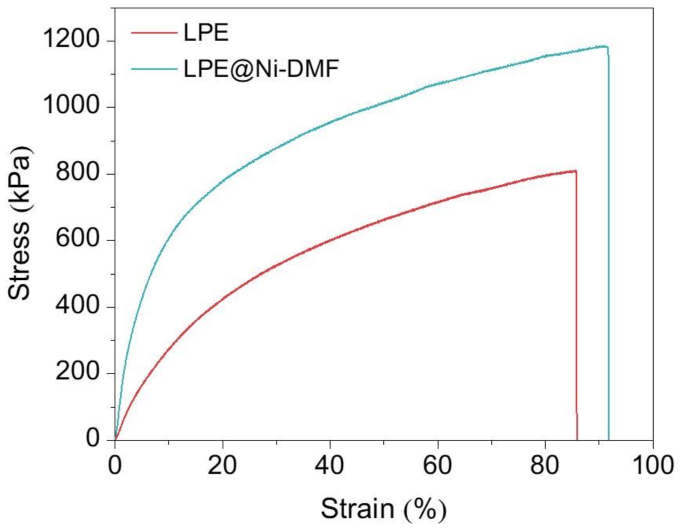

Supplementary Fig. 22 | Tensile modulus of elasticity of LPE and LPE@Ni-DMF membranes.

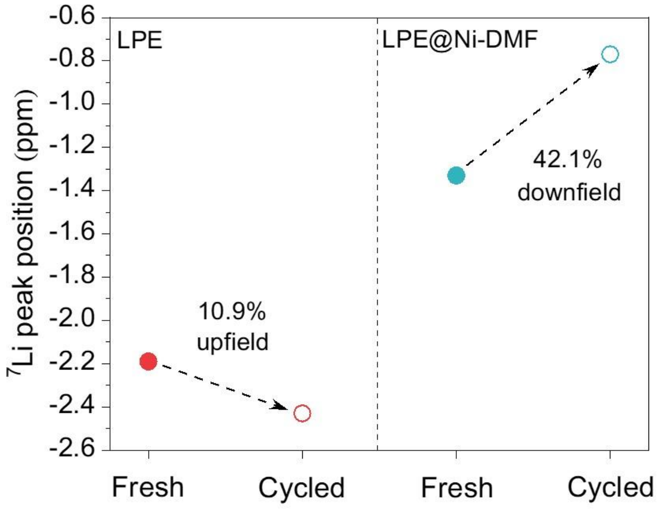

Supplementary Fig. 23 | Comparison of ^{7}Li singal shift of LPE and LPE@Ni-DMF at fresh and cycled states.

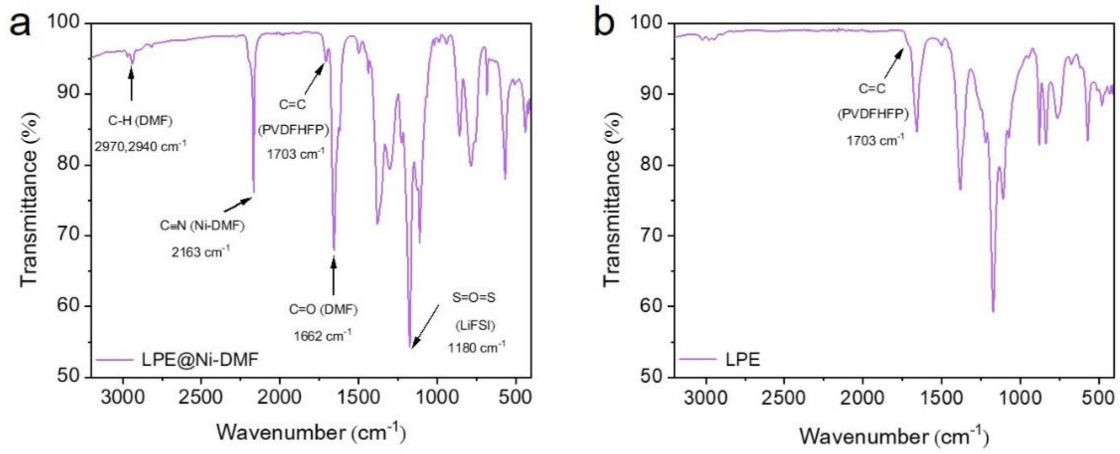

Supplementary Fig. 24 | ATR-FTIR spectra of LPE@Ni-DMF (a) and LPE (b).

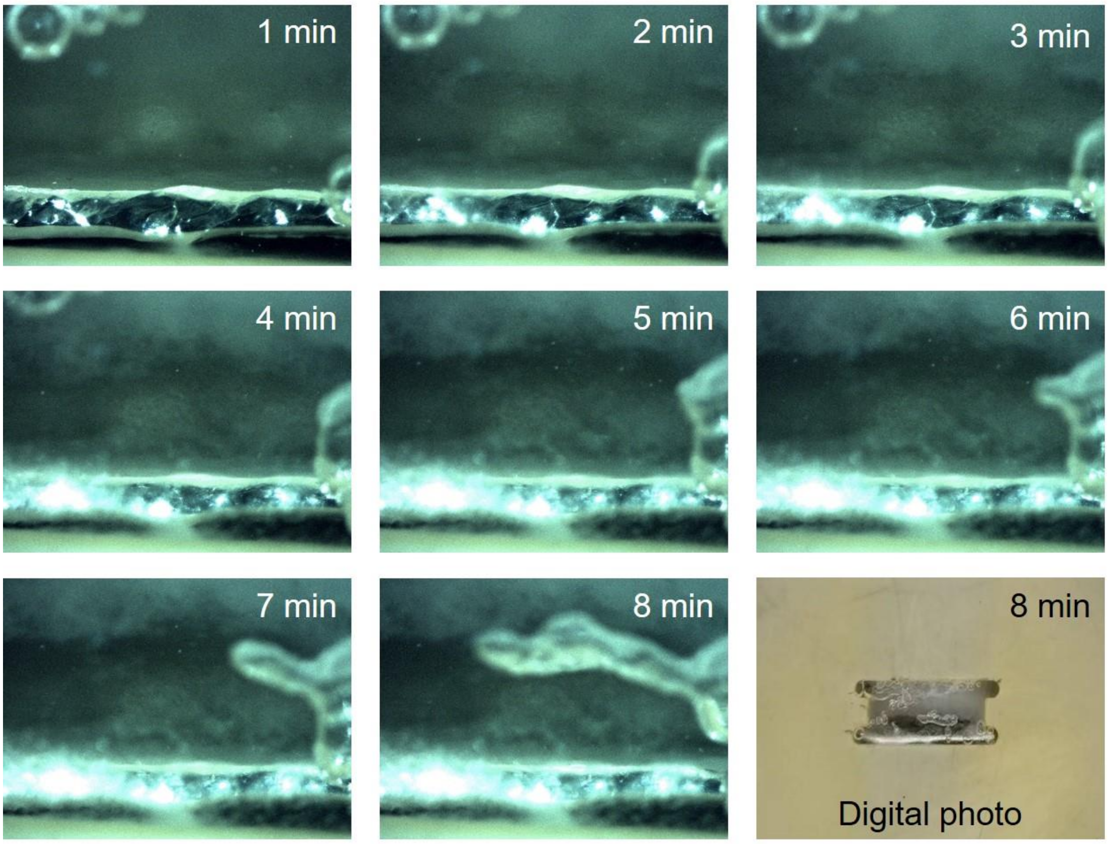

Supplementary Fig. 25 | In-situ optical microscope images of the reaction between Li metal and DMF.

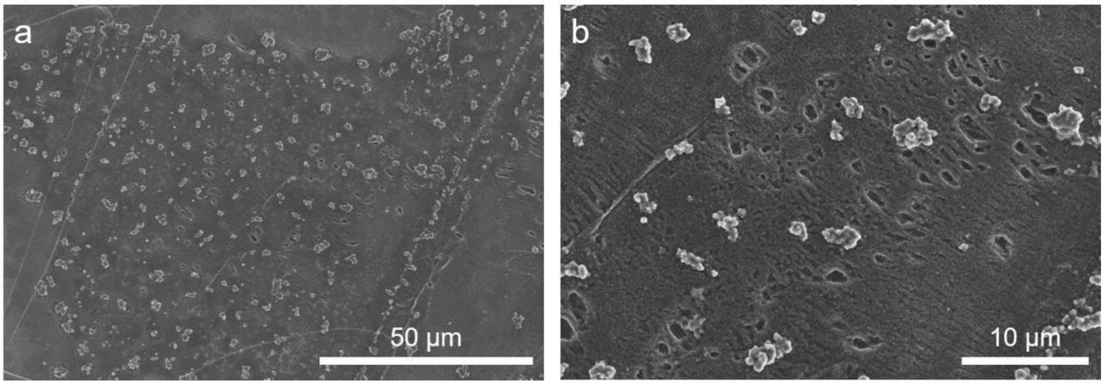

Supplementary Fig. 26 | SEM (a) and corresponding magnified (b) images of the Li metal corroded by DMF.

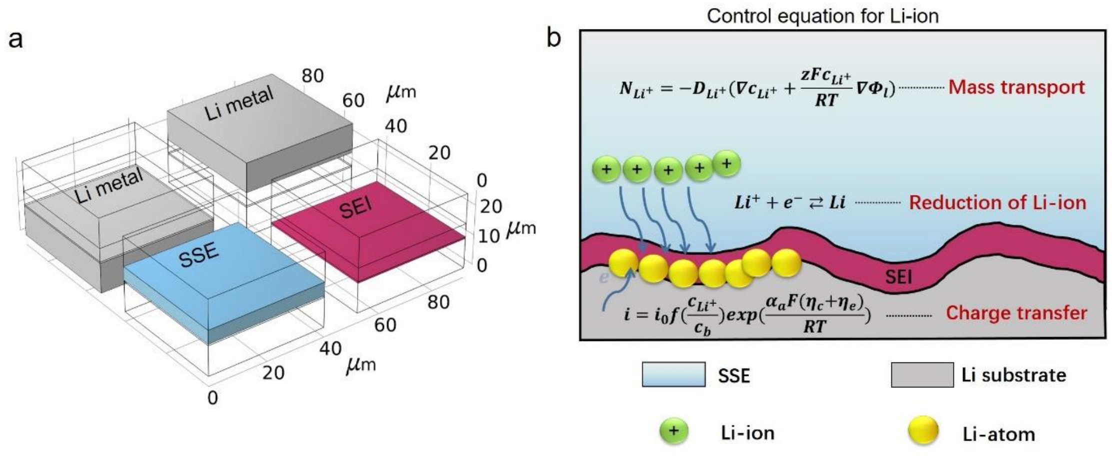

Supplementary Fig. 27 | Li||SSE||SEI||Li geometric model used in the finite element method simulation (a) and control equation for Li-ion behaviors (b).

The model used in the simulation for the electrochemical behaviors of Li^+ under different SEI, in which the ion conductivity is determined based on the results of corrosion simulation. The figure b illustrates the transport of Li^+ from the electrolyte to the SEI and the subsequent reduction to Li atom at the anode surface, which mainly includes two major processes: charge transfer and mass transport, and it could be expressed by the simplified reaction:

$$Li^+ + e^- \leftrightarrow Li \qquad (3)$$

The transport of Li^+ and charge transfer are respectively described by the Nernst-Planck equation and the Butler-Volmer equation (more details see simulation methods)[1,2]. Due to the large η in the Li metal anode, the Butler-Volmer equation can be simplified to the charge transfer equation depicted in the figure:

$$i \approx i_0 \left[\exp\left(\frac{\alpha_a F\eta}{RT}\right)\right] = f\left(\frac{c_{Li^+}}{c_b}\right) \exp\left(\frac{\alpha_a F(\eta_c+\eta_e)}{RT}\right) \qquad (4)$$

Where $f\left(\frac{c_{Li^+}}{c_b}\right)$ is the function of Li concentration gradient near the Li anode surface, η_c and η_e are the concentration polarization and electrochemical reaction polarization, respectively[3]. Thus, this figure depicts the numerical correlation among the local deposition rate, concentration gradient near the anode surface, as well as of polarization.

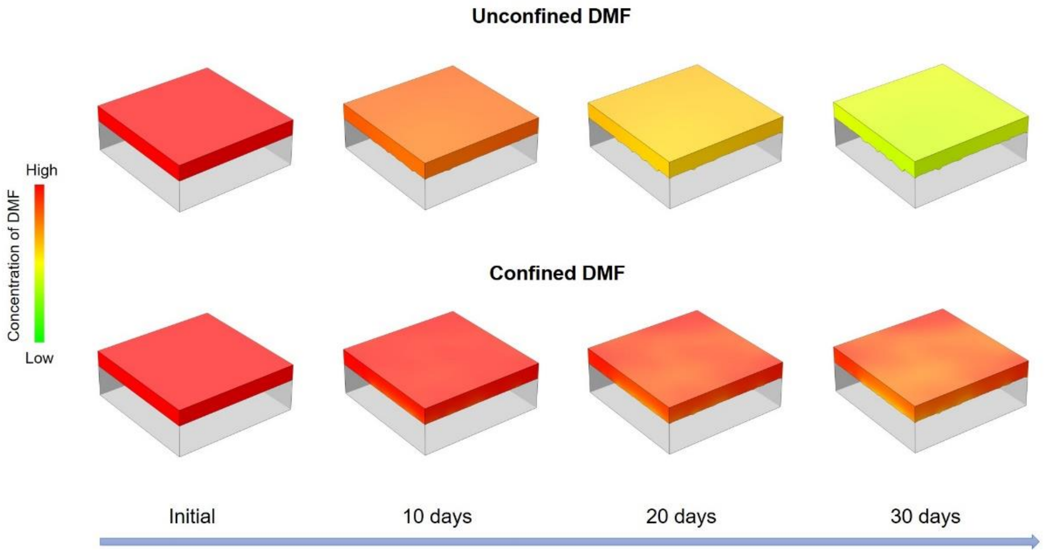

Supplementary Fig. 28 | Concentration evolution of DMF under an applied external electric field in LPE (unconfined DMF) and LPE@Ni-DMF (confined DMF).

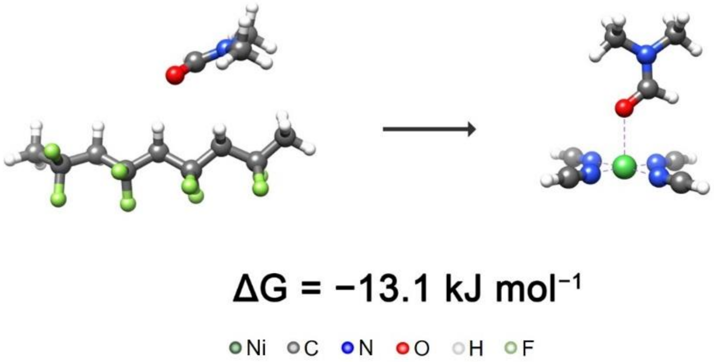

Supplementary Fig. 29 | Calculated adsorption Gibbs free energy change of DMF adsorbed on PVDF and Ni^{2+} site.

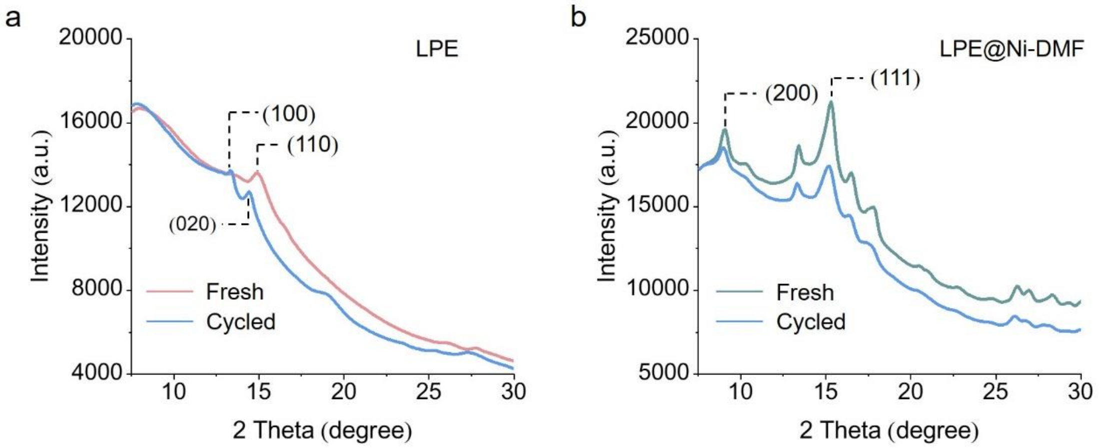

Supplementary Fig. 30 | Fresh and cycled diffraction patterns of (a) LPE and (b) LPE@Ni-DMF extracted from 2D wide-angle X-ray scattering.

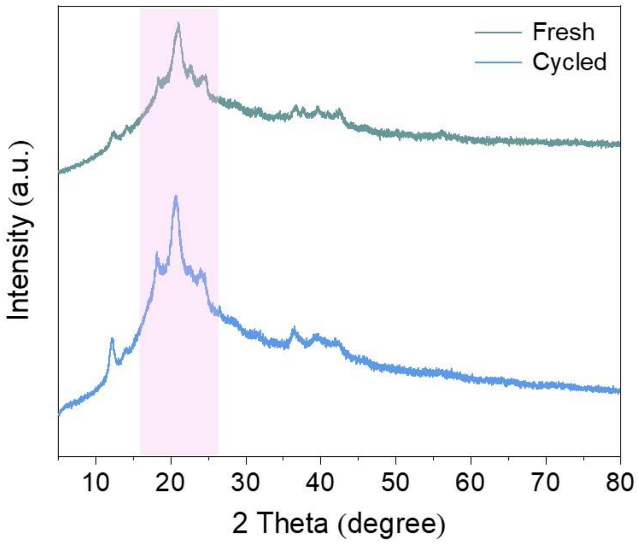

Supplementary Fig. 31 | XRD patterns of LPE@Ni-DMF before and after cycling.

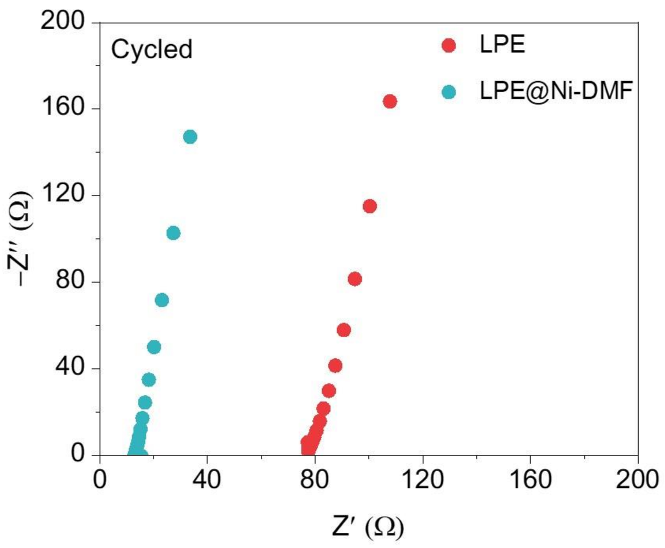

Supplementary Fig. 32 | Nyquist plots of cycled LPE and LPE@Ni-DMF at room temperature.

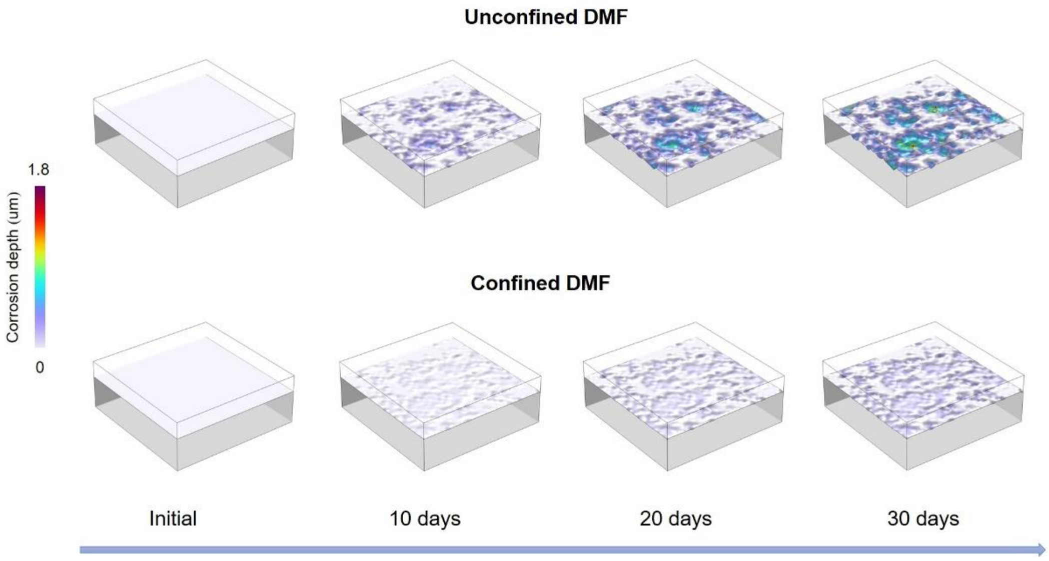

Supplementary Fig. 33 | Corrosion depth evolution of Li metal anode using LPE (unconfined DMF) and LPE@Ni-DMF (confined DMF).

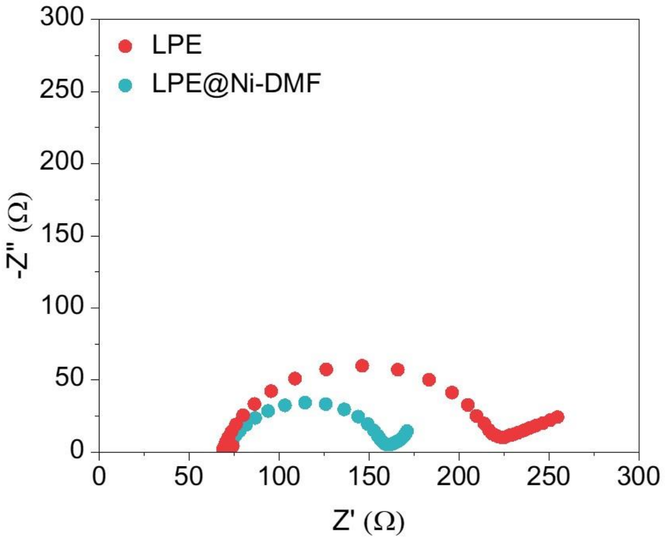

Supplementary Fig. 34 | Nyquist plots of Li|LPE|Li and Li|LPE@Ni-DMF|Li cells after cycling.

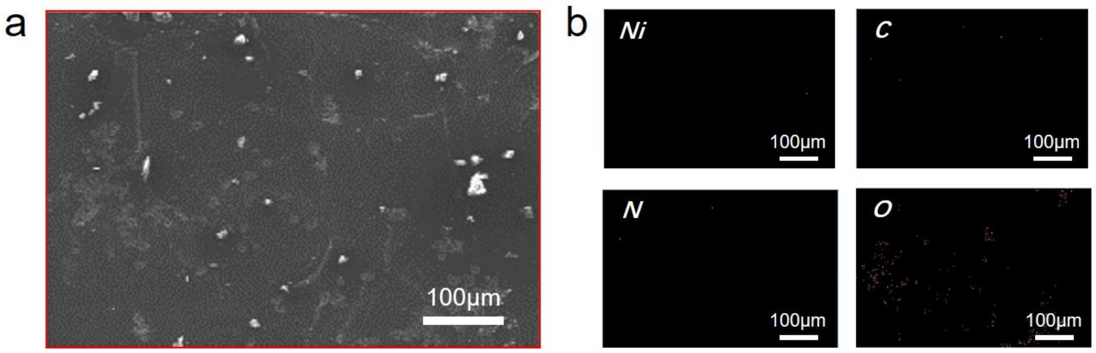

Supplementary Fig. 35 | Morphology and element characterization of cycled Li metal. (a) SEM image of the Li metal surface after Li|LPE@Ni-DMF|Li symmetric cell test at room temperature (20 hours of cycling at 0.1 mA cm^{-2} with capacity of 0.1 mAh cm^{-2} per half cycle) and (b) corresponding EDS mappings.

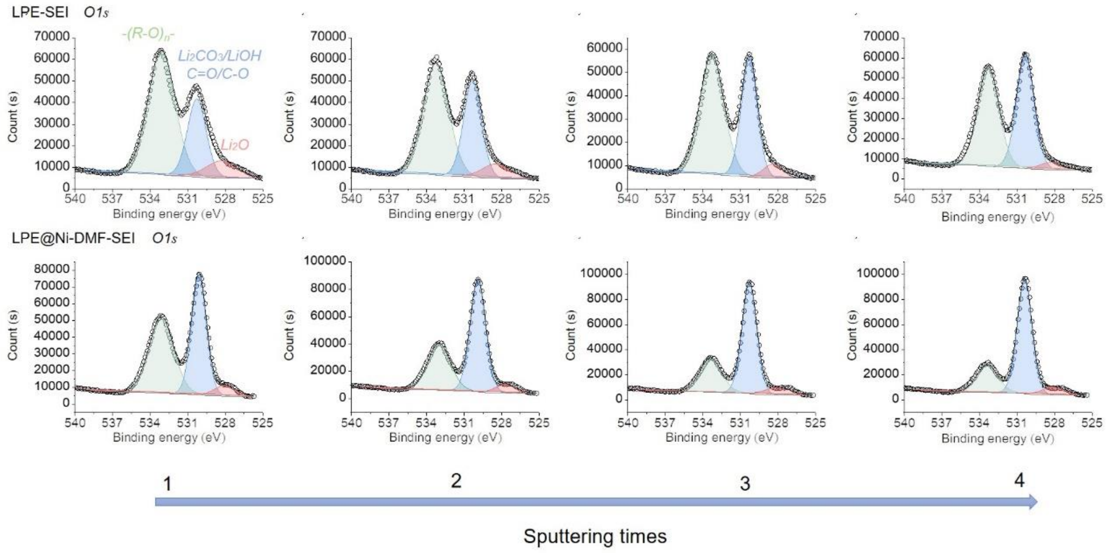

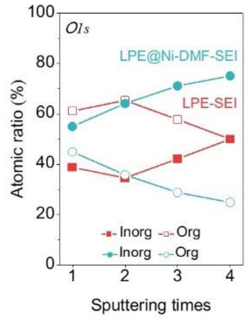

Supplementary Fig. 36 | X-ray photoelectron spectroscopy *O1s* depth profiles (by sputtering for different times) of cycled Li metal anode using LPE and LPE@Ni-DMF and corresponding atomic ratio change.

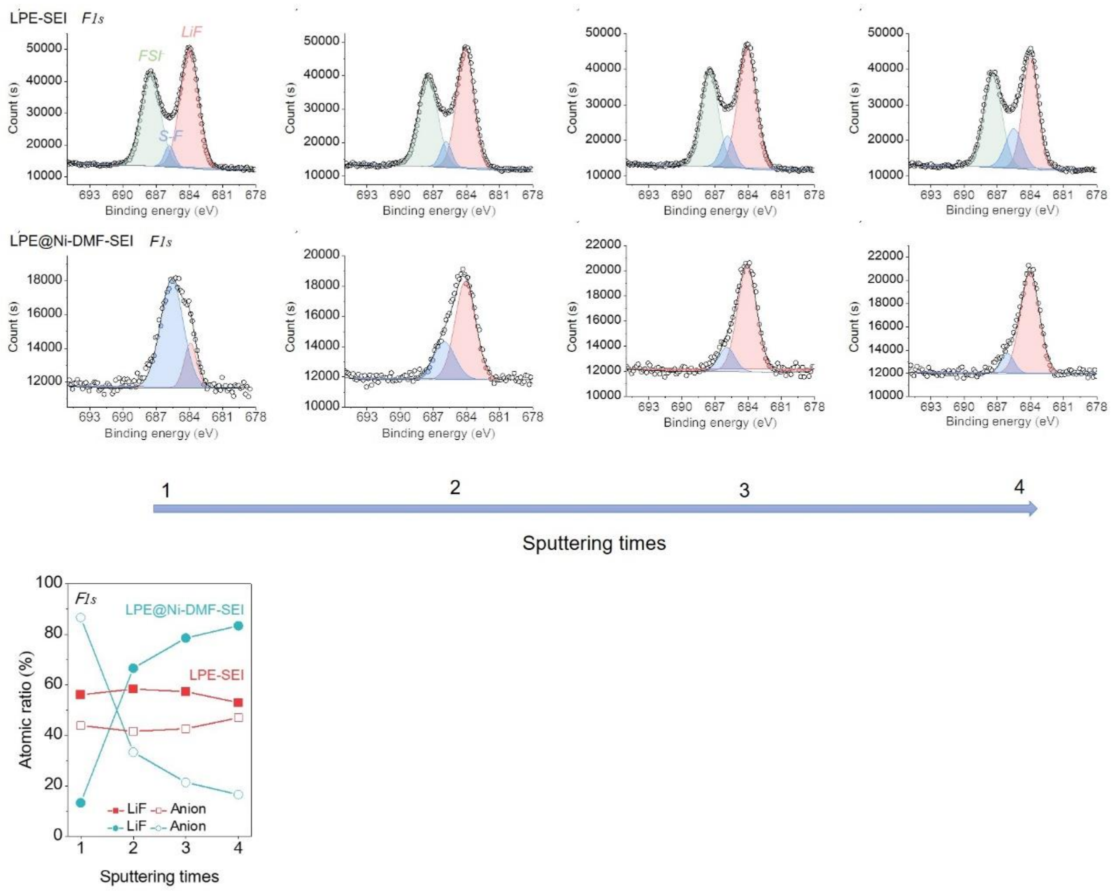

Supplementary Fig. 37 | X-ray photoelectron spectroscopy *F1s* depth profiles (by sputtering for different times) of cycled Li metal anode using LPE and LPE@Ni-DMF and corresponding atomic ratio change.

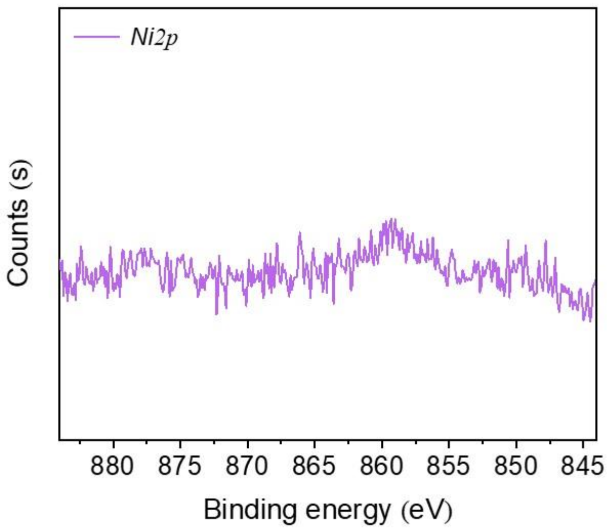

Supplementary Fig. 38 | *Ni2p* XPS spectrum of the Li metal surface after Li|LPE@Ni-DMF|Li symmetric cell test at room temperature (20 hours of cycling at 0.1 mA cm^{-2} with capacity of 0.1 mAh cm^{-2} per half cycle).

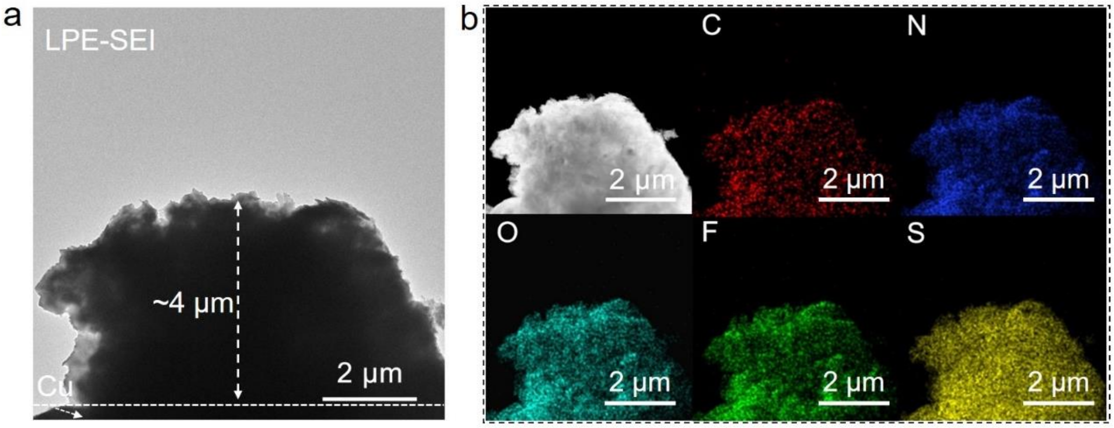

Supplementary Fig. 39 | Cryogenic transmission electron microscopy image (a) and corresponding element mappings (b) of LPE-SEI.

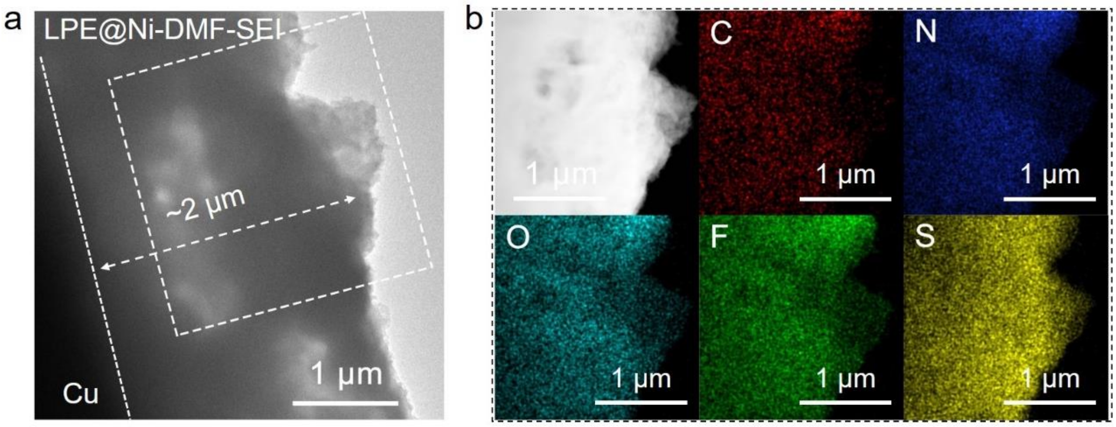

Supplementary Fig. 40 | Cryogenic transmission electron microscopy image (a) and corresponding element mappings (b) of LPE@Ni-DMF-SEI. The element mappings were conducted in the area surrounded by the white-dotted square in Supplementary Fig. 40a.

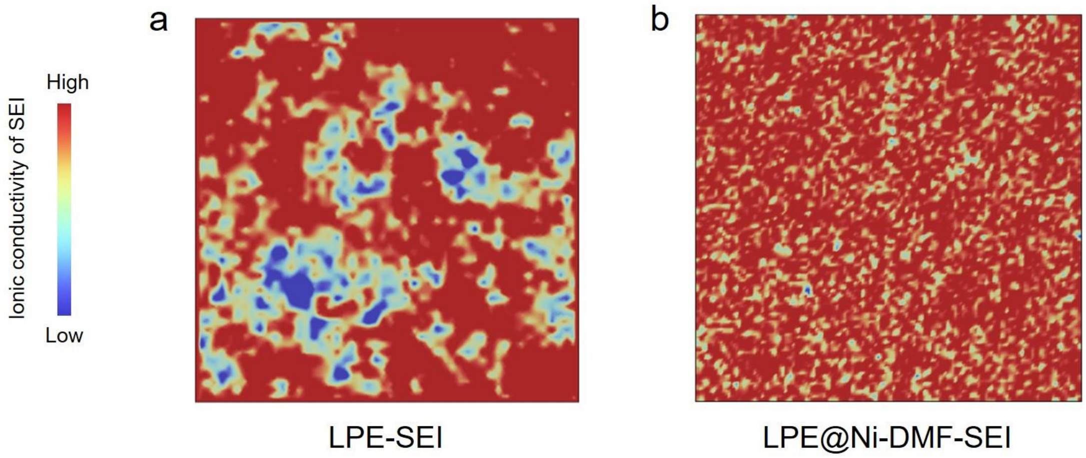

Supplementary Fig. 41 | Ionic conductivity distribution of LPE-SEI (a) and LPE@Ni-DMF-SEI (b) for the simulation of Li^+ electrochemical behaviors.

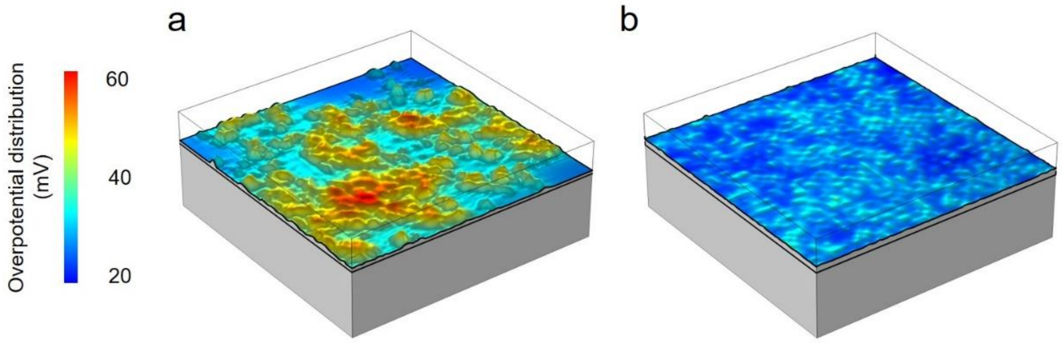

Supplementary Fig. 42 | Overpotential distribution on Li metal anode surfaces using LPE (a) and LPE@Ni-DMF (b).

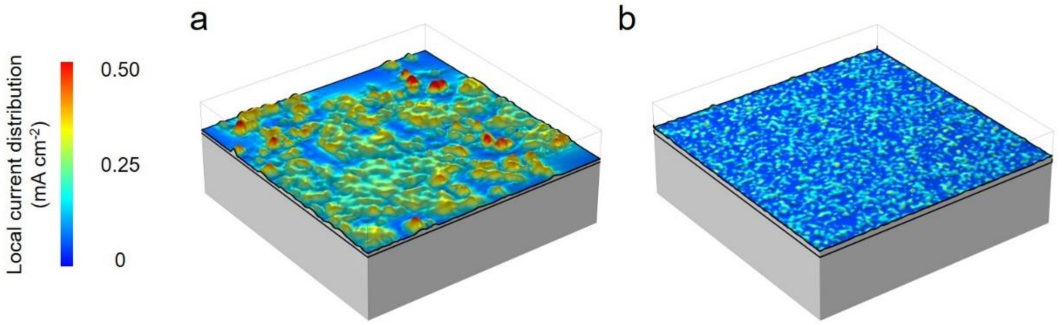

Supplementary Fig. 43 | Local current distribution on Li metal anode surfaces using LPE (a) and LPE@Ni-DMF (b).

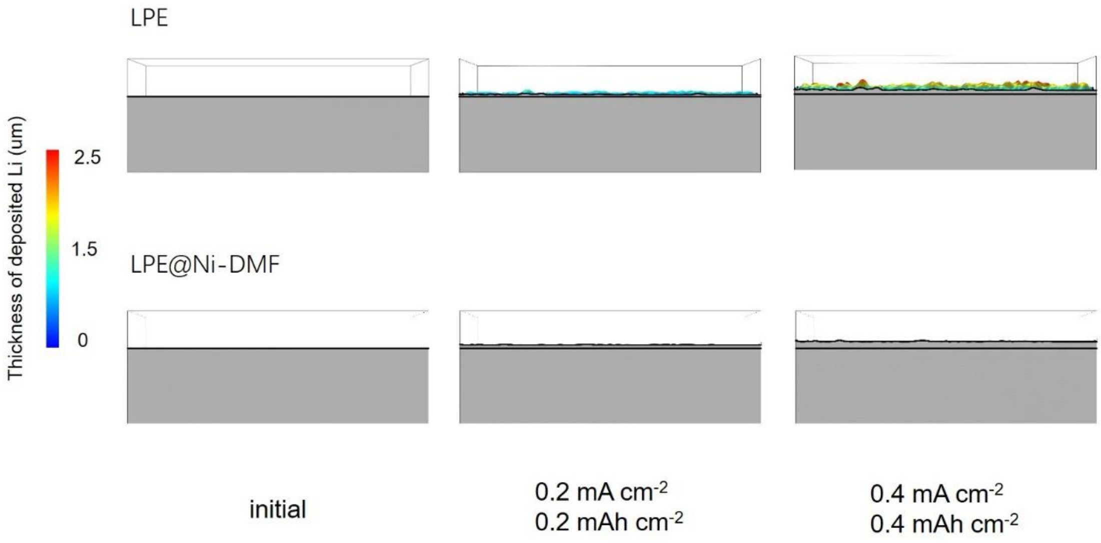

Supplementary Fig. 44 | Morphological evolution of Li plating under increased current densities using LPE and LPE@Ni-DMF.

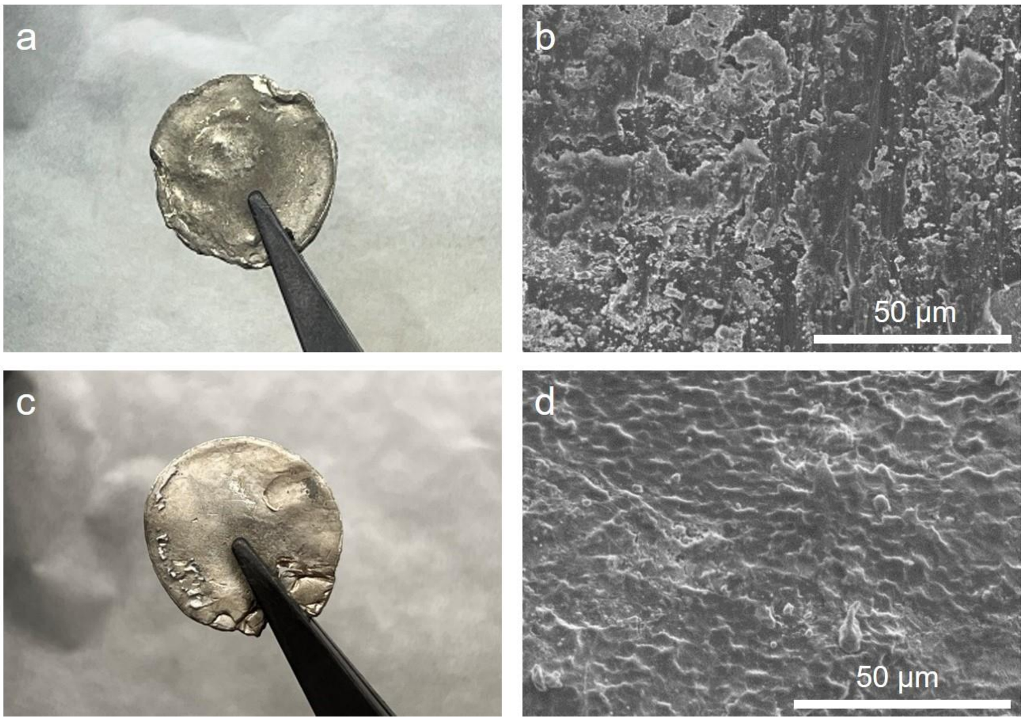

Supplementary Fig. 45 | Surface morphology of cycled Li metal anode using LPE (a) and LPE@Ni-DMF (c) and corresponding SEM images (b and d).

Bulk and dead Li deposits are observed in terms of LPE electrolyte. However, the Li deposits when using LPE@Ni-DMF electrolyte are almost flat and such morphology is consistent with its long cycle life in Li||Li symmetric cells.

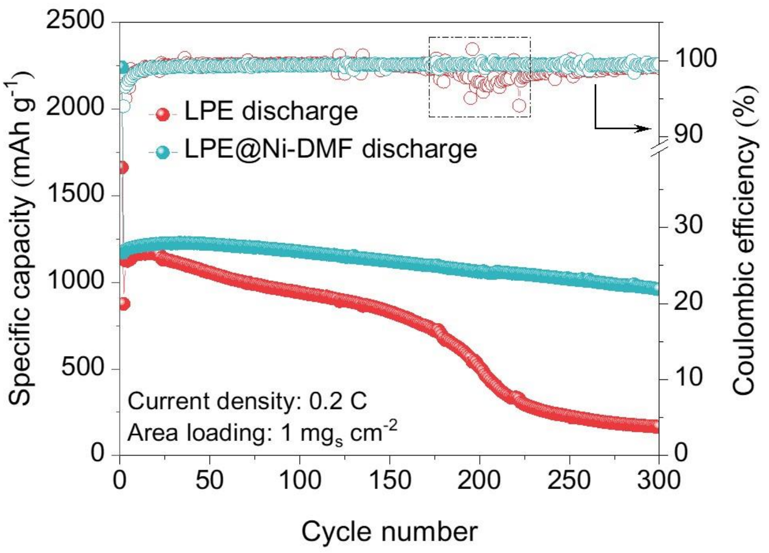

Supplementary Fig. 46 | Cycling performance of Li|LPE|SPAN and Li|LPE@Ni-DMF|SPAN coin cells at 0.2 C in the voltage range from 1 ~ 3 V.

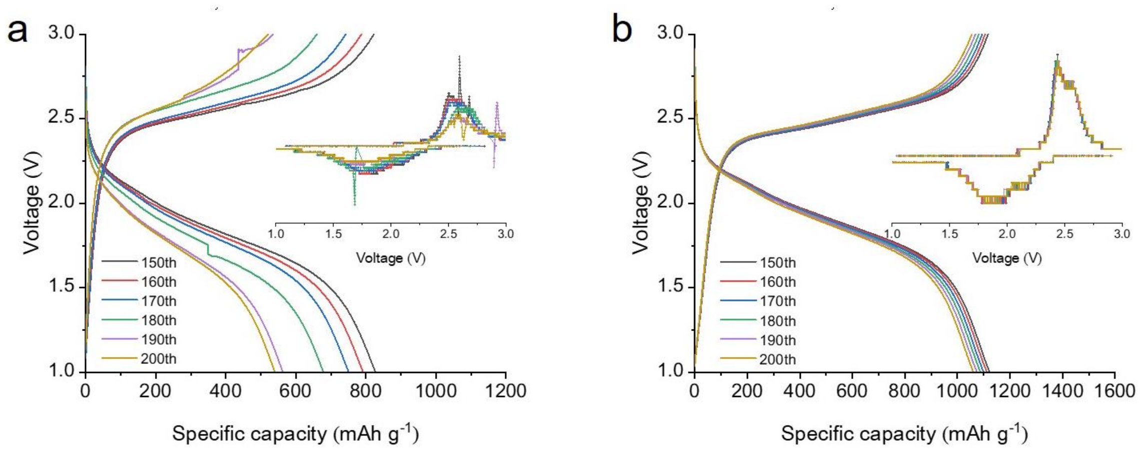

Supplementary Fig. 47 | Charge–discharge curves of Li|LPE|SPAN (a) and Li|LPE@Ni-DMF|SPAN (b) cells at the 150th, 160th, 170th, 180th, 190th, and 200th cycles. The figures inserted depict the corresponding dQ/dV results. Both cells were tested at 0.2 C in the voltage range from 1 ~ 3 V.

As one of the most common failures of solid-state batteries, "short circuit" can be categorized as "hard short circuit" or "soft short circuit" (also known as micro-short circuit). In the case of a "hard short", the voltage drops dramatically during the charging process and the battery is unable to recover, which is the most common short circuit phenomenon. In contrast, the "soft short" phenomenon is often observed in solid-state batteries, where the battery voltage is dynamically fluctuated but does not rise during charging, and the battery is able to recover from the short circuit. The voltage fluctuation is mainly caused by an uneven local electric field distribution due to lithium dendrite growth. This results in a decrease in Coulombic efficiency and a subsequent severe drop in capacity. As demonstrated in **Supplementary Fig. 47a**, the phenomenon of voltage

fluctuation is evident during the charging process, particularly for the 150th, 190th, and 200th cycles. This is further supported by the dQ/dV curves, which clearly show a significant raise or drop in potential. In contrast, the phenomenon of voltage fluctuation is not apparent in the Li|LPE@Ni-DMF|SPAN cell (**Supplementary Fig. 47b**), which demonstrates that our strategy can create a stable interface between LPE@Ni-DMF and Li metal, preventing micro-short circuits.

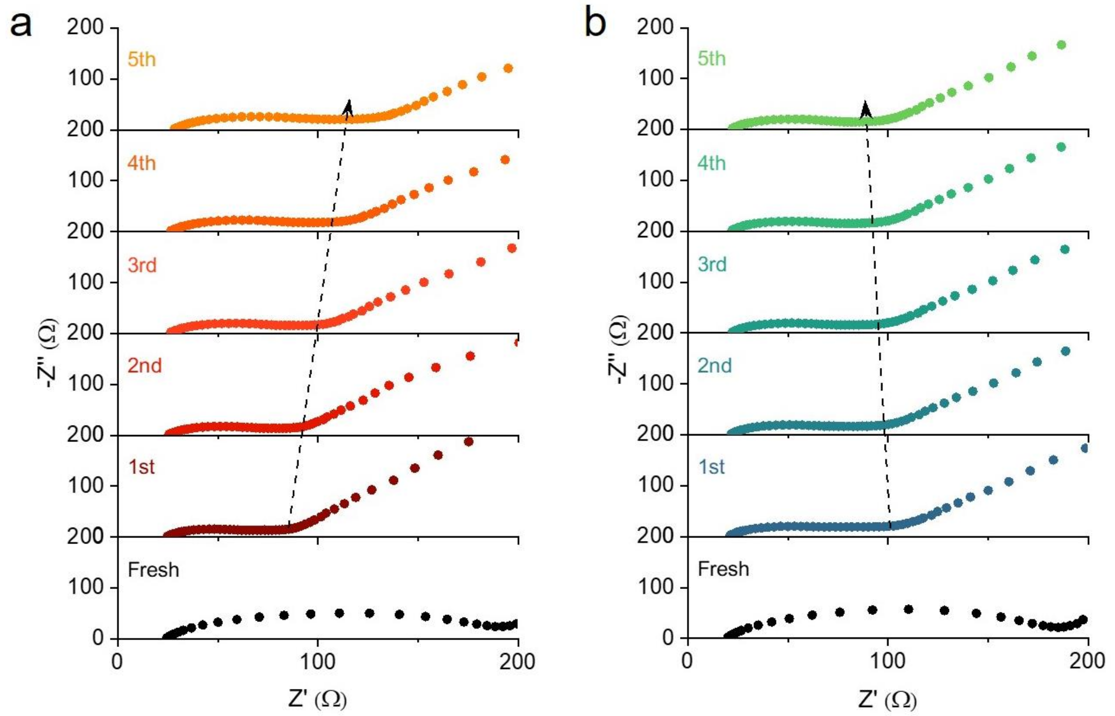

Supplementary Fig. 48 | Nyquist plots of (a) Li|LPE|SPAN and (b) Li|LPE@Ni-DMF|SPAN coin cells during first 5 cycles.

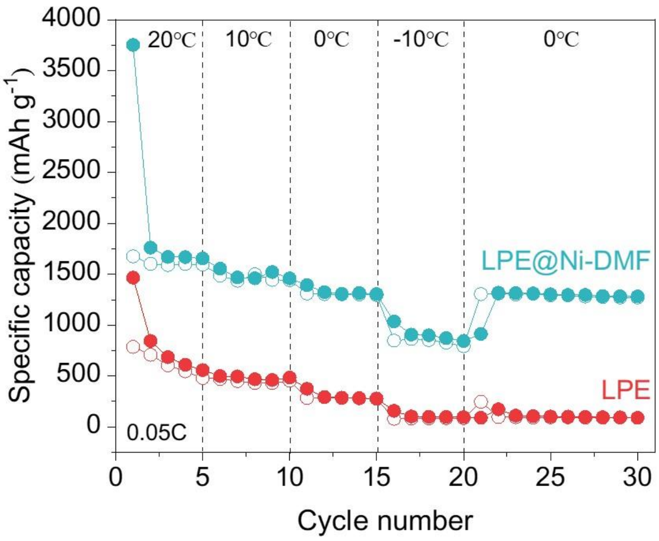

Supplementary Fig. 49 | Temperature-dependent cycling performance of Li|LPE|SPAN and Li|LPE@Ni-DMF|SPAN coin cells at 0.05 C in the voltage range from 1 ~ 3 V.

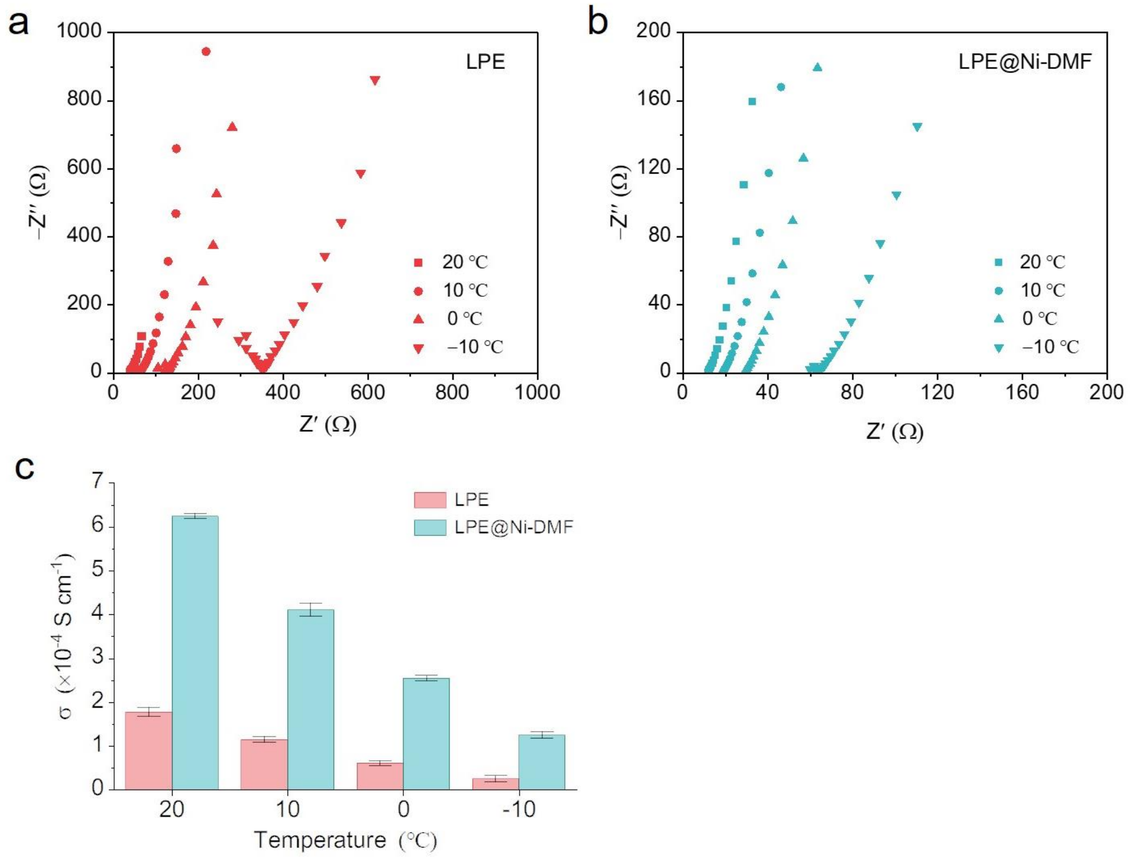

Supplementary Fig. 50 | Temperature-dependent Nyquist plots of (a) LPE and (b) LPE@Ni-DMF and corresponding ionic conductivities (c).

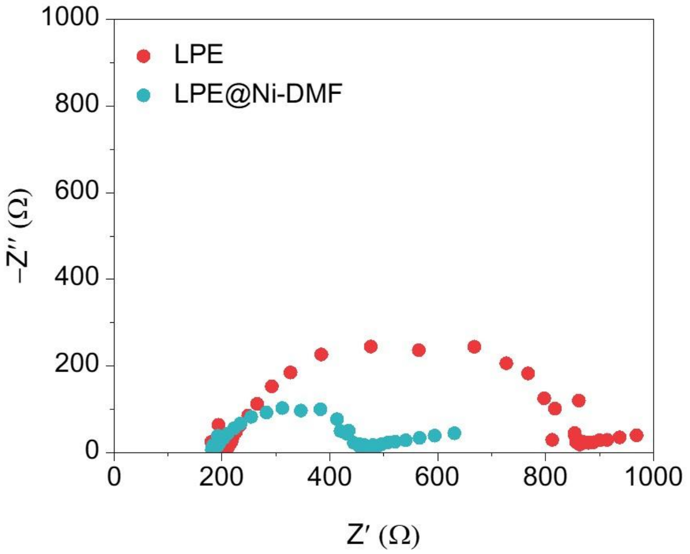

Supplementary Fig. 51 | Nyquist plots of Li|LPE|SPAN and Li|LPE@Ni-DMF|SPAN coin cells after cycling at 0 °C.

Supplementary Table 1 | Comparison of ionic conductivity (σ) and Li^+ transference number (t_{Li^+}) between this work and those in previous reports.

Sample name	σ (S cm^{-1})	t_{Li^+}	ref.
PVDF-LiFSI[a]	1.18×10^{-4}	/	4
PMLSE[b]	2.0×10^{-4}	0.62	5
HSE-etched bmLLZO30[c]	4.5×10^{-4}	/	6
LATP-PVDF-Li[d]	2.44×10^{-4}	0.52	7
PHLC (20% CAP)[e]	1.25×10^{-4}	0.49	8
S-LHCE[f]	2.7×10^{-4}	0.72	9
PVDF-DMF-LiFSI[g]	3.0×10^{-4}	0.44	10
PVDF-LPPO[h]	4.84×10^{-4}	0.47	11
LPE@Ni-DMF	6.5×10^{-4}	0.71	This work

[a] PVDF-LiFSI is the abbreviation of polymer-based electrolyte comprised of poly(vinylidene difluoride) (PVDF) and LiFSI. [b] PMLSE is the abbreviation of PVDF-HFP/MOF composite gel/LLZN nanowires solid electrolyte. [c] HSE-etched bmLLZO30 is the abbreviation of hybrid solid electrolytes comprised of 70 wt% PVDF and 30 wt% ball-milled and CF_4 plasma etched LLZO filler. [d] LATP-PVDF-Li is the abbreviation of composite solid electrolyte comprised of LATP powders, PVDF, and LiTFSI. [e] PHLC (20% CAP) is the abbreviation of polymer electrolyte comprised of PVDF-HFP, LiTFSI, and cellulose acetate propionate with a weight ratio of 20% with respect to PVDF-HFP. [f] S-LHCE is the abbreviation of solidified localized high-concentration electrolyte. [g] PVDF-DMF-LiFSI is the abbreviation of solid polymer electrolyte comprised of PVDF, trace DMF solvent, and LiFSI. [h] PVDF-LPPO is the abbreviation of lithium phenyl phosphate grafted onto PVDF.

Supplementary References

1. Liu, Y. et al. Insight into the critical role of exchange current density on electrodeposition behavior of lithium metal. *Adv. Sci.* **8**, 2003301 (2021).
2. Xu X. et al. Diffusion limited current density: A watershed in electrodeposition of lithium metal anode. *Adv. Energy Mater.* **12**, 2200244 (2022).
3. Xu X. et al. Role of Li-ion depletion on electrode surface: Underlying mechanism for electrodeposition behavior of lithium metal anode. *Adv. Energy Mater.* **10**, 2002390 (2020).
4. Zhang, X. et al. Self-suppression of lithium dendrite in all-solid-state lithium metal batteries with poly(vinylidene difluoride)-based solid electrolytes. *Adv. Mater.* **31**, 1806082 (2019).
5. Sun, J. et al. Facilitating interfacial stability via bilayer heterostructure solid electrolyte toward high-energy, safe and adaptable lithium batteries. *Adv. Energy Mater.* **10**, 2000709 (2020).
6. Lee, M. J. *et al.* Interfacial barrier free organic-inorganic hybrid electrolytes for solid state batteries. *Energy Storage Mater.* **37**, 306–314 (2021).
7. Gao, C. et al. Cellulose acetate propionate incorporated PVDF-HFP based polymer electrolyte membrane for lithium batteries. *Compos. Commun.* **33**, 101226 (2022).
8. Liu, L. et al. Synergistic effect of lithium salts with fillers and solvents in composite electrolytes for superior room-temperature solid-state lithium batteries. *ACS Appl. Energy Mater.* **5**, 2484–2494 (2022).
9. Liu, Q. et al. Polymer electrolytes based on interactions between [solvent-Li^+] complex and solvent-modified polymer. *Energy Storage Mater.* **51**, 443–452 (2022).
10. Mi, J. et al. Topology crafting of polyvinylidene difluoride electrolyte creates ultra-long cycling high-voltage lithium metal solid-state batteries. *Energy Storage Mater.* **48**, 375–383 (2022).
11. Xu, S. et al. Decoupling of ion pairing and ion conduction in ultrahigh-concentration electrolytes enables wide-temperature solid-state batteries. *Energy Environ. Sci.* **15**, 3379–3387 (2022).
