# Supplementary material for: A locally solvent-tethered polymer electrolyte for long-life lithium metal batteries
Source: Nat Commun. 2024 May 9;15:3914. doi: 10.1038/s41467-024-48078-7 (PMC11082227; doi:10.1038/s41467-024-48078-7)
Supplement: Supplementary file 3 — Description of Additional Supplementary Files [file 41467_2024_48078_MOESM3_ESM.pdf]

Description of Additional Supplementary Files

Supplementary Movie 1: The split rendering map of LPE@Ni-DMF membrane using X-ray computed tomography.

Supplementary Movie 2: The three-dimensional reconstruction of LPE@Ni-DMF membrane.

Supplementary Movie 3: The evolution of SEI thickness in a Li|LPE|Li symmetric cell using finite element method simulation.

Supplementary Movie 4: The evolution of SEI thickness in a Li|LPE@Ni-DMF|Li symmetric cell using finite element method simulation.

Supplementary Movie 5: The Li deposition behavior in a Li|LPE|Li symmetric cell using finite element method simulation.

Supplementary Movie 6: The Li deposition behavior in a Li|LPE@Ni-DMF|Li symmetric cell using finite element method simulation.
